# Cognitive performance and brain dynamics during walking with a novel bionic foot: A pilot study

**DOI:** 10.1371/journal.pone.0214711

**Published:** 2019-04-03

**Authors:** Kevin De Pauw, Pierre Cherelle, Bruno Tassignon, Jeroen Van Cutsem, Bart Roelands, Felipe Gomez Marulanda, Dirk Lefeber, Bram Vanderborght, Romain Meeusen

**Affiliations:** 1 Research Group Human Physiology, Faculty of Physical Education and Physical Therapy, Vrije Universiteit Brussel, Brussels, Belgium; 2 Strategic Research Program ‘Exercise and the Brain in Health & Disease: the added value of Human-Centered Robotics’, Vrije Universiteit Brussel, Brussels, Belgium; 3 Department of Mechanical Engineering, Faculty of Applied Sciences, Vrije Universiteit Brussel and Flanders Make, Brussels, Belgium; 4 Artificial Intelligence Lab, Faculty of Sciences and Bioengineering Sciences, Vrije Universiteit Brussel, Brussels, Belgium; The Ohio State University, UNITED STATES

## Abstract

**Objectives:**

The objectives are to determine neural dynamics during gait using electro-encephalography and source localization, and to investigate the attentional demand during walking in able-bodied individuals, and individuals with an amputation.

**Materials & methods:**

Six able-bodied individuals conducted one experimental trial, and 6 unilateral transtibial and 6 unilateral transfemoral amputees performed 2 experimental trials; the first with the prosthesis currently used by the subjects and the second with a novel powered transtibial prosthesis, i.e. the Ankle Mimicking Prosthetic foot 4.0. Each experimental trial comprised 2 walking tasks; 6 and 2 minutes treadmill walking at normal speed interspersed by 5 minutes of rest. During 6 minutes walking the Sustained Attention to Response (go-no go) Task, which measures reaction time and accuracy, was performed. Electro-encephalographic data were gathered when subjects walked 2 minutes. Motor-related cortical potentials and brain source activity during gait were examined. Normality and (non-) parametric tests were conducted (p<0.05).

**Results and discussion:**

In contrast to transtibial amputees, transfemoral amputees required more attentional demands during walking with Ankle Mimicking Prosthetic foot 4.0 compared to the current passive prosthetic device and able-bodied individuals (reaction time and accuracy: p≤0.028). Since risk of falling is associated with altered attentional demands, propulsive forces of the novel device need to be better controlled for transfemoral amputees. No motor-related cortical potentials at Cz were observed in transfemoral amputees walking with the novel prosthesis, whereas motor-related cortical potentials between transtibial amputees and able-bodied individuals during walking at normal speed did not differ. The first positive electro-physiological peak deflection appeared during toe-off phase and showed higher activity within the underlying brain sources in transtibial amputees walking with Ankle Mimicking Prosthetic foot 4.0 compared to able-bodied individuals. The required higher neural input to accomplish the same physical activity compared to able-bodied individuals is possibly due to the limited acclimation period to the novel device and consequently increased afferent sensory feedback for postural control.

## Introduction

Persons with an amputation strongly depend on prostheses to cope with daily activities. Reality is that prostheses available on the market are still equipped with technology that is 30 years old, mostly non-articulated passive mechanisms. The limitations of passive lower limb prostheses compared to an actual human leg reduces gait performance, induces compensatory movements and increases fatigue, as well as the incidence for injuries at the level of contact between the socket and the residual limb. However, with the past decades’ technological advances in mechatronics, computational power and research in mechanical engineering, prosthetics have become a source of interest from roboticists. World-wide, engineers strive at improving prosthetics’ design and functionalities with the aim of optimising comfort and dexterity during daily activities. Although most of these advances are still on a research level, their results show a preview of the upwards potential future prosthetics hold for amputees [1]. Recently, a novel bionic foot, the Ankle Mimicking Prosthetic-foot or AMPfoot 4.0, has been developed at the Vrije Universiteit Brussel (Belgium). AMPfoot 4.0 is a new type of energy efficient below knee prosthesis providing improved passive adaptive and propulsion characteristics to its wearer. A previous paper of De Pauw et al. [2] observed that the metabolic cost of AMPfoot 4.0 during walking is higher compared to the current prosthetic foot and able-bodied individuals. Nevertheless, both transtibial and transfemoral amputees (TTA and TFA, respectively) recognize the added value of the locking-unlocking spring set mechanism used in its design. A major concern of the novel bionic foot is that suboptimal functioning reduces comfort during walking. The latter might happen in TFA, since they do not lift the heel of the prosthetic device high enough to trigger the energy release of the spring set.

Since the central and peripheral nervous system play a fundamental role in human gait and other daily activities [3], one of the opportunities and future challenges is the implementation of neuroprosthetics, meaning that the prosthetic device is controlled by electrical signals from the muscle or the brain. The acquired electrical signal is then used to optimize the movement of an artificial limb [4]. A first step towards non-invasive brain-computer interfaces of lower-limb prostheses is determining the supraspinal control of human locomotion. A recent paper of Luu et al. [5] clearly outlined that a better understanding of the neural dynamics of walking has potential implications for the use in brain-machine interfaces. Electro-encephalography (EEG) is of special interest since it has a very high temporal resolution. EEG recording during walking is challenging due to noise, but several research groups managed to gather reliable EEG data during gait [6,7,8].

EEG derived averaged time-locked movement-related events, or movement-related cortical potentials (MRCPs) reflect electro-cortical activity related to the planning and execution of movements. The MRCP of a voluntary movement consists of a Bereitschaftspotential or ‘pre-motor potential’, followed by a motor potential [9]. The MRCP amplitude typically lies between 5 and 30 μV, and only occurs within the delta frequency range [10]. The main generators of the MRCP are motor cortices, i.e. the supplementary motor area, primary motor, premotor and cingulate cortices [11]. Brain areas responsible for the negative movement-related cortical potential are the supplementary motor area and primary motor cortex [12]. In a previous experiment of Knaepen et al. [7] temporal and spatial characteristics of averaged electro-cortical activity during treadmill walking were investigated in healthy subjects using EEG. As a result, characteristic temporal patterns of positive and negative potentials, similar to MRCPs, were observed over the cortical leg representation area (EEG central electrode position at the midline: Cz). For advancing towards brain-machine interfaces for lower-limb prostheses, it is of importance to determine whether brain dynamics is altered in persons with an amputation compared to able-bodied individuals, since an amputation impairs sensory feedback and alters central nervous system reorganization.

Human movement and postural control during walking and other daily activities also require attentional resources. In neuro-physiological research dual-task paradigms are used to investigate the attentional demand of walking [13] and are vital for determining neural dynamics during human movement. The dual-task paradigm requires an individual to simultaneously perform two tasks that interfere with each other, meaning that the same attentional resources are used for both tasks [14].

The goal of this pilot study is two-fold: First, neural dynamics during gait are examined using EEG extracted MRCPs, as well as their brain sources of MRCPs. Second, the attentional demand during walking in able-bodied individuals and individuals with a unilateral transtibial (TTA) and transfemoral (TFA) amputation with current, traditional passive and novel bionic prostheses is investigated. The hypothesis is that impaired sensory feedback and central nervous system reorganization following amputation alters motor preparation and command between subject groups. It is hypothesized that subjects with an amputation require more attentional demands during walking compared to able-bodied individuals. We also assume that subjects with an amputation require less attentional demands during walking with their current prosthesis, to which subjects are accustomed to, compared to the novel prosthetic device. Able-bodied individuals were included to investigate the resemblance in attentional demands and EEG MRCPs during walking with individuals with an amputation.

## Materials and methods

### Subjects

Three subject groups were included in the current study, i.e. 6 able-bodied individuals (5 men, 1 woman, age: 26 ± 5yrs, height: 1.75 ± 0.10m and weight: 72 ± 12kg), 6 subjects with a unilateral transtibial amputation (TTA: 6 men, all left amputated, K-level 4, age: 54 ± 14yrs, height: 1.76 ± 0.08m and weight: 80 ± 13kg) and 6 with a unilateral transfemoral amputation (TFA: 5 men, 1 woman, 5 right, 1 left amputated, K-level 4, age: 53 ± 14yrs, height: 1.79 ± 0.09m and weight: 89 ± 16kg). Recruitment of the subjects took place in September and October 2016. Able-bodied individuals were recruited at the university, whereas TTA and TFA were recruited from an orthopaedical center VIGO, located in Wetteren (Belgium). The current prosthetic knees of TFA were passive mechanical knees from Ottobock (types 3R15 or 3R80). The prosthetic passive foot of all subjects with an amputation was Solid-Ankle Cushion-Heel-foot (Ortho Europe), a prosthetic foot that dampens the impact forces during heel strike.

Subjects were provided written and oral information about the experimental procedures and potential risks before giving written informed consent to participate in this study. The experiment and consent procedure were approved by the institutional medical ethics commission of the University Hospital Brussel and Vrije Universiteit Brussel (Belgium) (B.U.N.143201526629) and the Federal Agency for Medicines and Health Products (reference: AFMPS/80M0641).

### Protocol

**Fig 1:**
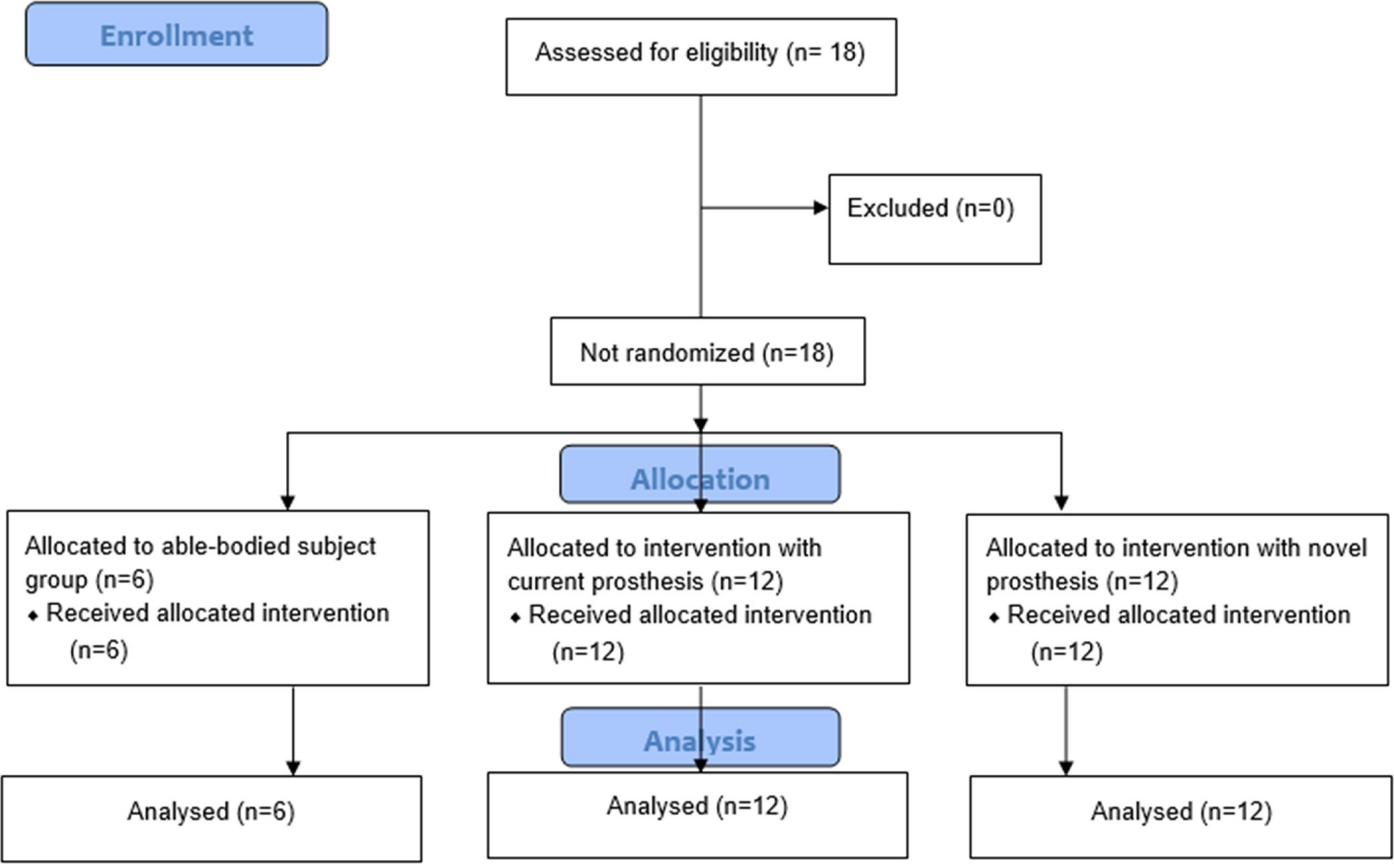
Presents the Consort flow diagram (enrolment, allocation and analysis). After the familiarization trial, two experimental trials were conducted; the first trial was performed with the current prosthesis and the second trial with the AMPfoot (4.0) (Fig 2). The AMPfoot 4.0 was fitted by a professional prosthetist using individualized connectors and shanks. Furthermore, subjects were familiarized with the novel device for about 15 to 30 minutes preceding experimental trial 2. The AMPfoot 4.0 design is also based on previous research conducted on the AMPfoot 2 [15] and 3 [16]. However, it is important to note that in contrast with its preceding designs, the AMPfoot 4.0 does not provide active propulsion at push-off.

The experiment was executed in November and December 2016. Subjects with an amputation visited the orthopaedical center VIGO 3 times with at least one day of rest in between trials; 1 familiarization trial and 2 experimental trials (Fig 1). Able-bodied individuals visited the laboratory only once (Fig 1); familiarization was performed preceding the experimental trial. A familiarization trial was included to accustom subjects to the experimental protocol. Additionally, biometrical measurements (as well as shoe and head size) were obtained. The size of the EEGcap was selected according to head size and the shoe size was important to provide customized insoles with a force-sensing resistor (406, Interlink Electronics).

**Fig 2:**
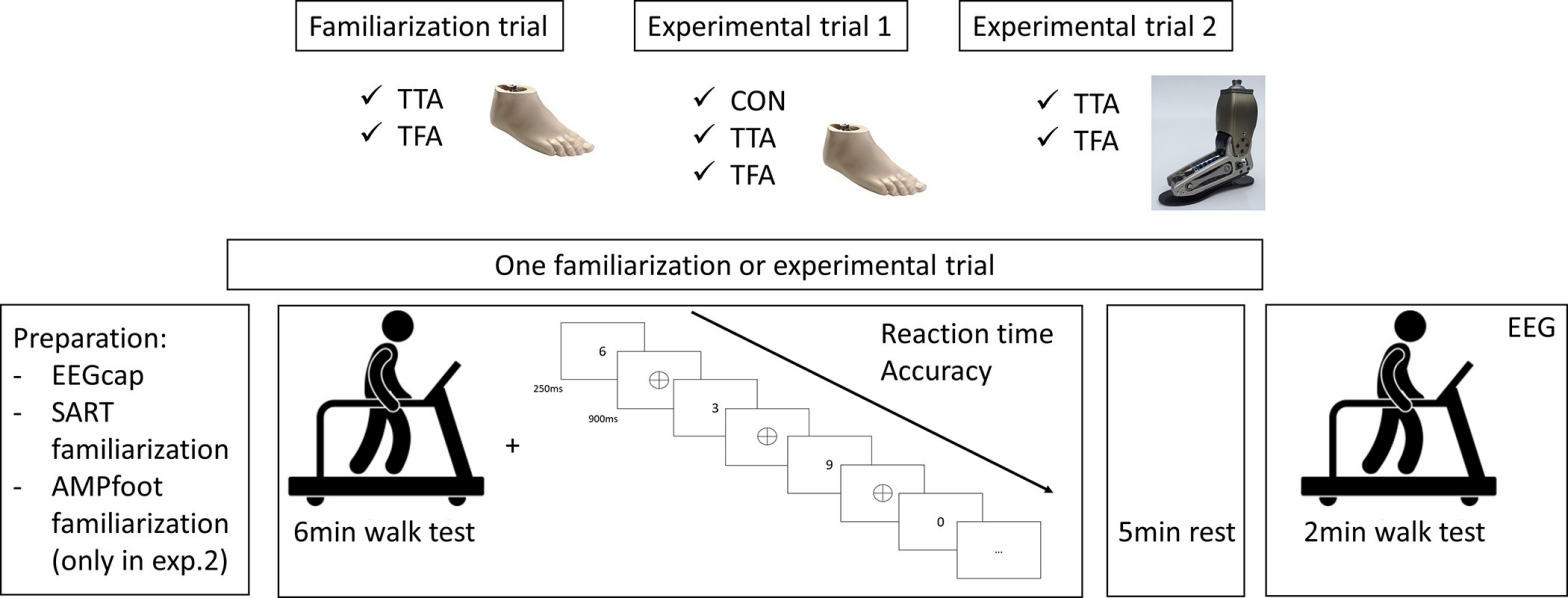
Experimental design and protocol. Dual-task walking was performed during the 6 minutes walk test. The cognitive task consisted of ten digits [0 1 2 3 4 5 6 7 8 9] randomly displayed for 250ms on a screen positioned in front of the subject followed by a 900ms mask. After a 5 minutes rest period subjects conducted the 2 minutes walk test and EEG was reapplied on the subjects’ head.

During the stance phase of walking, the AMPfoot 4.0 stores elastic potential energy by means of a spring set. During the swing phase, the locking mechanism unlocks to free the ankle movement. Parallel springs external to the stance system are then activated to reset the foot to its initial position. From that moment, the device is ready for a new step.

Each experimental trial started with familiarizing the attentional (cognitive) task, i.e. the Sustained Attention to Response Task (duration: 4.3 minutes) [17]. The visual stimuli were displayed in black and white in the middle of a laptop screen that was placed in front of the treadmill (about 0.8m from the subject and at a fixed height). The cognitive task consists of 10 digits [0 1 2 3 4 5 6 7 8 9]. Each digit is displayed 25 times in a randomized order. Five different font sizes (48, 72, 94, 100 or 120 point) were used, which corresponds to a height varying between 12-29mm. Each digit was presented on the screen for 250ms followed by a 900ms mask (Symbol of a ring with a diagonal cross in the middle; diameter ring: 29mm). Subjects were asked to sustain attention throughout the test, not to talk and to respond as fast as possible to all digits (go), except digit 3 (no go). The subjects had to respond using the thumb on a button of a rectangular device that fitted well in the right hand. The first Sustained Attention to Response Task (familiarization trial) was performed in standing position. The second Sustained Attention to Response Task was performed during the 6 minutes walking task and commenced after 1 minute of treadmill walking. Reaction time (ms) and accuracy (%) of the go as well as the accuracy of no go stimuli were registered during dual-task walking. For the analysis of accuracy datasets were divided in 5 time slots (4*1 minute and 1*20s; Accuracy1 - …—Accuracy5).

The walking task was 6 minutes treadmill walking, wherein, normal, comfortable speed, determined during the familiarization trial, was maintained for both experimental trials. Able-bodied individuals walked at a constant speed of 5.2km/h. The walking speed was limited for able-bodied individuals to 5.2km/h in order to avoid the transition from walking to running. TTA and TFA walked at a self-selected speed of 4.1 ± 1.2km/h and 2.8 ± 0.9km/h, respectively. After the 6min treadmill walking task and a rest period of 5 minutes, subjects walked 2 minutes at self-selected speed. During the 2 minutes walking task EEG measurements were gathered. The same speed was maintained throughout the two experimental trials.

Preceding the 6min walking task thirty-two active silver/silver chloride electrodes were attached on the subjects’ head (Acticap, Brain Products, Munich, Germany) according to the 10–20 International System [18]. Impedance was kept below 10kΩ and baseline measures were collected before the 6min treadmill walking task. During the 6min walking task, the EEGcap was removed from the subjects’ head. The active electrodes contain an ultra-low noise pre-amplifier located inside the electrode, which leads to an attenuation of artifacts and signal noise resulting from high impedance electrodes and the skin, artifacts caused by electrode or cable movements, distorted signals or background noise. Different sizes of EEGcaps (54, 56, 58 or 60cm) were used according to the head size of the subject and connected to the amplifier BrainAMP DC (Brain Products, Munich, Germany) (digitization dept: 16 bit, high cutoff frequency: 1000Hz and resolution/unit: 0.1μV). The laboratory was darkened to reduce visual distraction and input. Subjects were also asked to focus on a small cross on the wall during the experiment (to reduce eye ball movement artifacts). Additionally, subjects were provided with earplugs to reduce sound artifacts.

Brain Vision Recorder and Brain Vision Analyzer (Version 2.0.4) were used to collect and (pre-)process the EEG datasets, respectively. Using visual inspection and statistical measures (e.g. kurtosis) bad channels were removed. In total, 8 channels (TP10 (n = 5), P7 (n = 2) and P3 (n = 1)) showed that noise level was substantially higher compared to the other channels. In only one dataset 2 electrodes needed to be removed, i.e. P3 and P7. Afterwards, removed channels were topographically interpolated (by spherical splines; order: 4; degree: 10; Lambda: 10^−5^). Data were re-referenced to an average reference (including interpolated bad channels) and down-sampled to 512Hz. Infinite Impulse Response Filters were set at 0.1Hz (high pass) and 30Hz (low pass). Raw data inspection was conducted. Since we isolate delta rhythms for the MRCP analysis, almost no manual artifact removal (solely severe muscle artifacts, electrode shifts) was needed. Periodically recurring artifacts (eye and muscle artifacts), were extracted from the data using Independent Component Analysis (Classic sphering, Extended Biased Infomax), a method used to estimate sources providing noisy measurements. Since average-referenced data were used for Independent Component Analysis, one channel was excluded from the analysis to optimally process independent components. Interpolated channels were also excluded from ICA. The main criteria to determine whether the independent component is brain-related or an artifact, are the scalp maps, the time course of the component as well as the activity power spectrum. Channel noise has been accounted for using scalp topography. If the weighting is set on a single channel, the independent component was removed from the dataset. After the Independent Component Analysis input data quality was controlled. The number of steps, i.e. the iterations of Independent Component Analysis algorithm until the algorithm has converged to a valid solution (av ± sd: 448 ± 17 steps) was smaller than the maximum number of steps (512 for Infomax), so requirements are met for Independent Component Analysis (i.e. clean input data). Regarding the number of restarts 10 restarts is common to get nice results and convergence. The current datasets needed 6 ± 2 restarts, which also indicates clean input data. Bad components were removed taking into account the time course, matrix of weights and topographies. Selected good independent components were back projected to the original EEG signals.

The force-sensing resistor was positioned under the foot of the subjects with an amputation, and in able-bodied individuals always under the right foot. Since the force-sensing resistor marks heel strike and is connected to EEG recording, it allows us to extract time-locked segments representing gait cycles. Heel strikes throughout the datasets were marked taking into account force-sensing resistor signals. Butterworth Zero Phase Filters were used on EEG data, and set at a high and low pass filter of 0.1 and 6Hz, respectively (slope: 48dB/oct). Every segment consisted of heel strike, stance phase, swing phase and again heel strike.

All electrodes underwent the same procedure of EEG (pre)processing to obtain MRCP data, but the focus was set at electrode Cz, since lower-extremity sensory-motor information processing occurs in the brain area located under Cz [19]. All segments (49 ± 21) were averaged and peak detection of the first positive (P1), the first negative (N1), the second positive (P2) and the second negative deflection (N2) were individually determined. Latencies (ms) and amplitudes (μV) of the different peaks were extracted from the individual EEG files.

To investigate the activity of the brain sources of these 4 MRCP deflections the source localization technique standardized low resolution brain electromagnetic tomography was conducted using the MNI-Average305-T1 anatomy. Text (.txt) files containing amplitude measures of 32 electrodes (columns) and 32 data points (rows) from each individual positive and negative peak deflection were extracted from Brain Vision Analyzer and inserted in the program standardized low resolution brain electromagnetic tomography. A transformation matrix (.spinv) file containing information of the electrode positions was constructed. Each .txt file was transformed to 1 cross spectrum (.crss) file and a standardized low resolution brain electromagnetic tomography (.slor) file in order to conduct further statistics.

### Statistical analysis

SPSS version 24.0 was used for the statistical analyses. Shapiro-Wilk normality tests of the different datasets were performed.

To determine the effect of AMPfoot on cognitive performance (reaction time) mixed ANOVAs were conducted (between groups TTA and TFA; within: prosthetic device and time). To compare data between the groups TTA and TFA with able-bodied individuals repeated-measures ANOVAs were performed for reaction time (between groups able-bodied individuals, TTA and TFA; within: time). If significant differences were found, post hoc comparisons (Bonferroni) were performed.

One-way ANOVAs were conducted to investigate differences in amplitude and latency between able-bodied individuals and TTA for the 4 MRCP deflections during gait. Additionally, within TTA paired samples T-tests were performed for each MRCP deflection. Hedges g effect sizes have also been determined. T statistics on log transformed standardized low resolution brain electromagnetic tomography data was also performed. The classical critical t value was determined (i.e. without smoothing), randomization (bootstrap with 5000 iterations) was performed and critical thresholds and p values were computed. When a voxel value exceeds the critical t value a significant difference in that brain area is detected. Statistical non-Parametric Mapping was applied for the correction of multiple comparisons.

For accuracy data Kruskal-Wallis H tests were performed between able-bodied individuals, TTA and TFA, as well as post hoc Mann-Whitney U tests. Within TTA and TFA Friedman ANOVAs were performed as well as Wilcoxon-signed rank tests. The critical alpha for all analyses was set at 0.05.

## Results

### Dual-task walking

**Fig 3:**
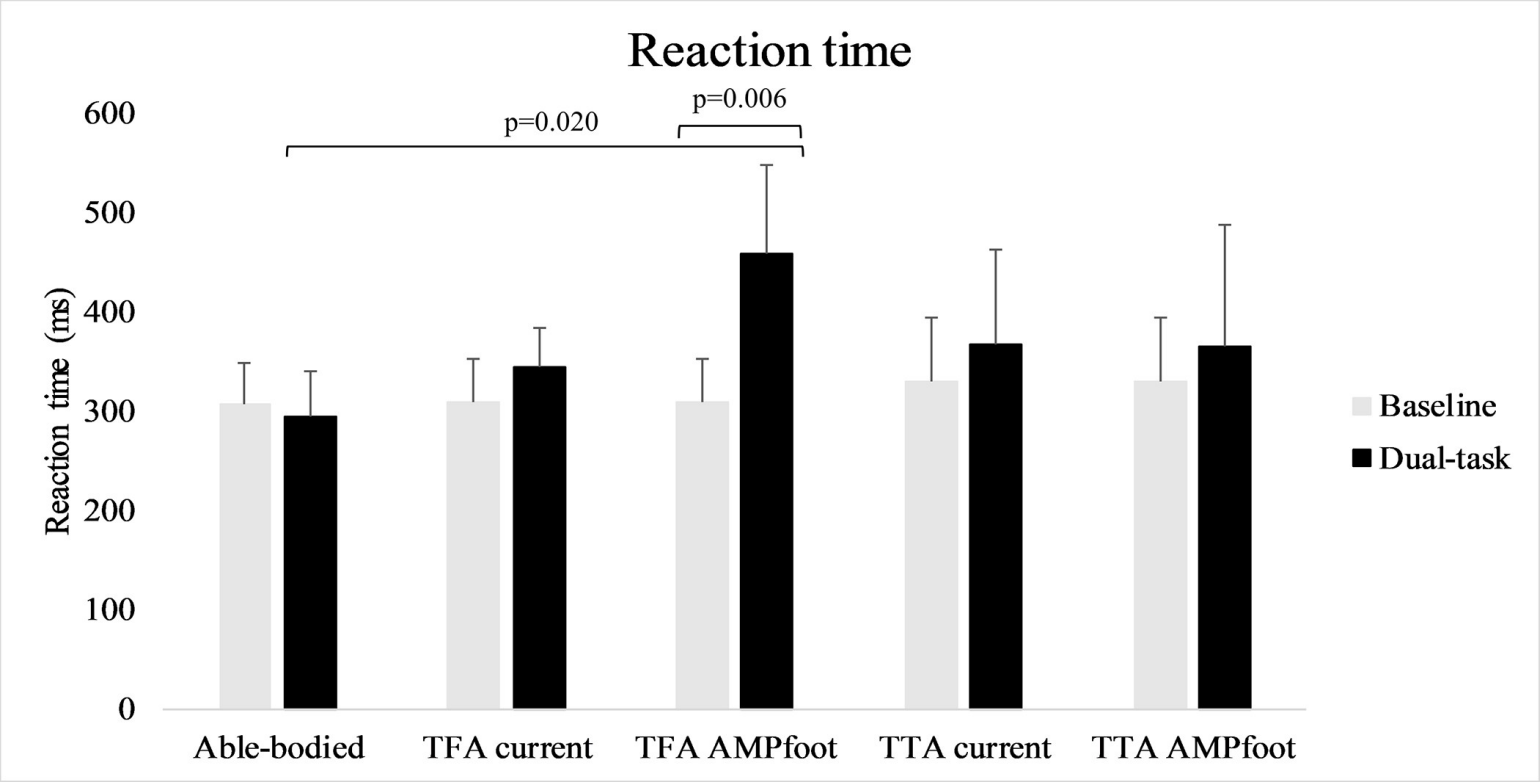
Shows reaction times (ms) of all subject groups at baseline and during dual-task walking.

During walking a main effect of prosthesis for reaction time was observed (F(1,10) = 15.650, p = 0.003), and an interaction effect prosthesis*groups (p = 0.002) was observed. Further repeated-measures ANOVA (within factors: time and prosthesis) revealed a main effect of prosthesis for TFA (F(1,5) = 21.056, p = 0.006) (Fig 3). Post hoc comparisons revealed significant higher reaction times for TFA with AMPfoot compared to able-bodied individuals (p = 0.020) (Fig 3).

**Fig 4:**
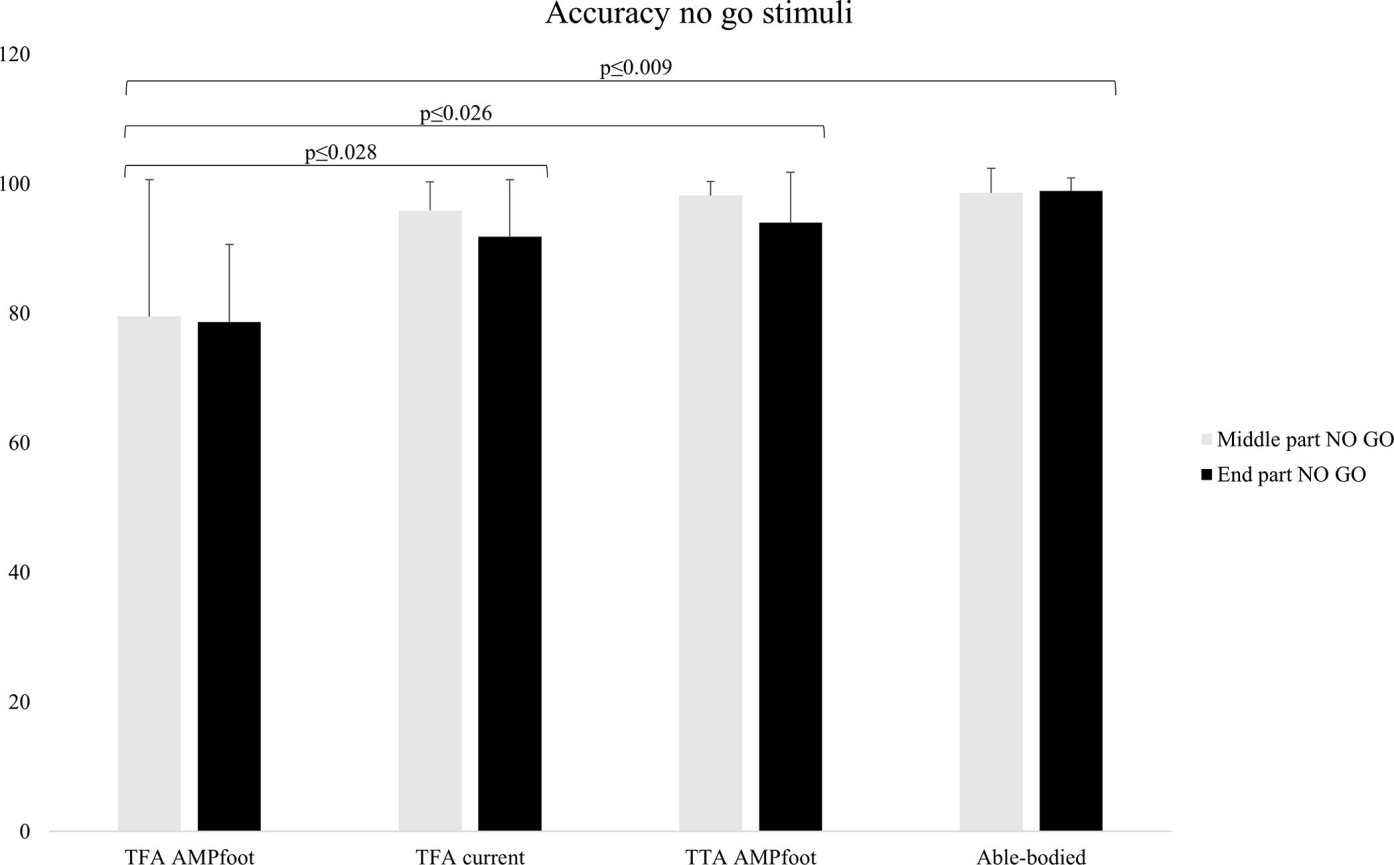
Accuracy of the no go stimuli during the middle part (Accuracy3) and at the end of the cognitive task (Accuracy5) for TFA walking with AMPfoot compared to TFA walking with the current prosthesis, TTA walking with the AMPfoot and able-bodied walking.

During walking with AMPfoot significant accuracy differences of the no go stimuli at the middle (Accuracy3) and end (Accuracy5) part of the cognitive task (χ^2^(2) = 9.554; p = 0.008 and χ^2^(2) = 10.144; p = 0.006, respectively) were observed. The significant differences observed via Mann-Whitney U tests are displayed in Fig 4. No other significant differences were observed for accuracy data.

### Motor-related cortical potentials and standardized low resolution brain electromagnetic tomography

**Fig 5:**
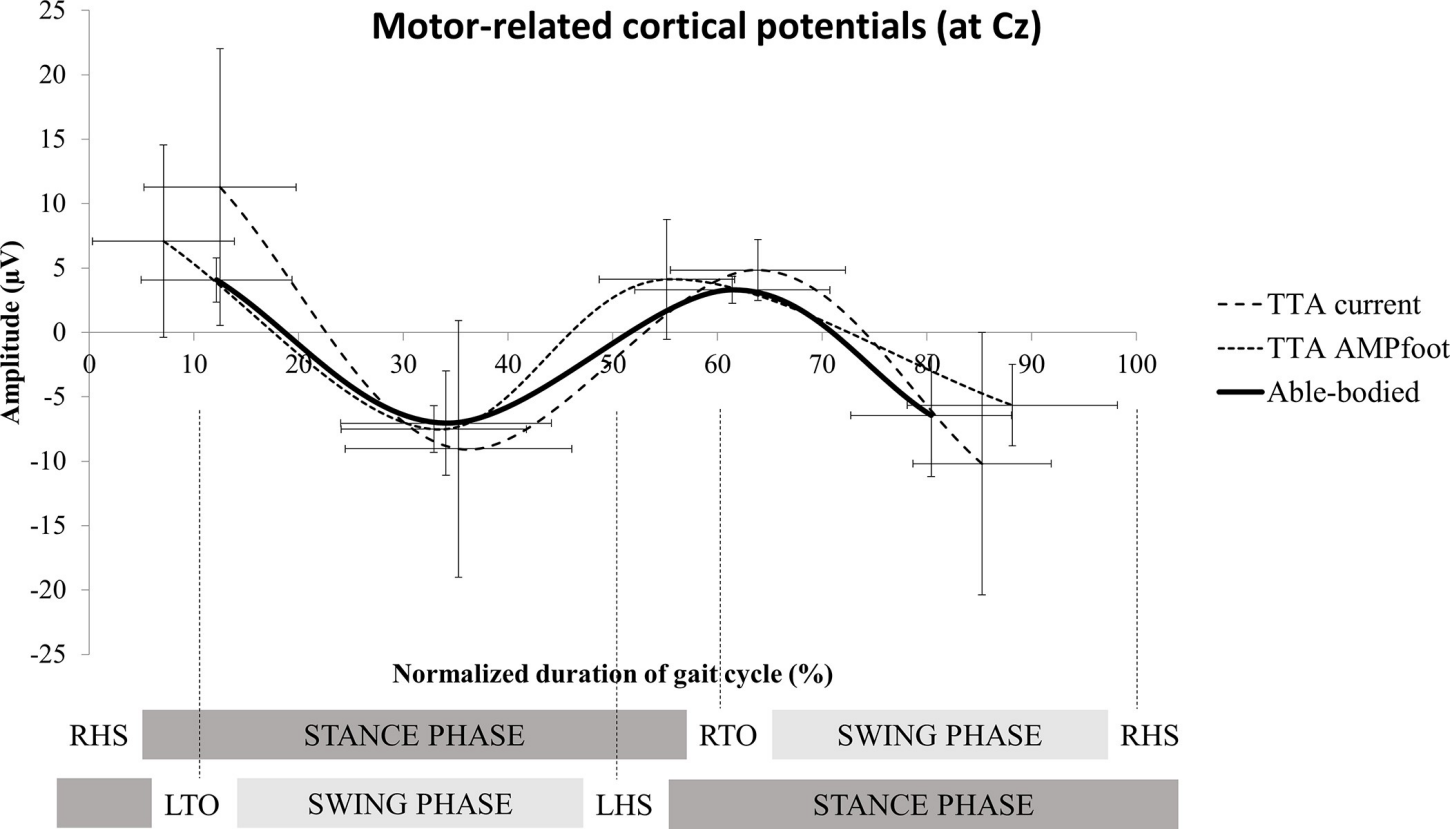
Shows a smoothed scatterplot showing EEG waveform (two positive deflections P1 and P2, and two negative deflections N1 and N2) recorded over the Cz electrode during an averaged gait cycle of able-bodied individuals and TTA with current and novel prosthetic devices. **Standard deviations are visualized for both x and y axes. An example of gait cycle phases and events is shown below the graph (here: force-sensing resistor placed in right insole).** RHS: right heel strike, RTO: right toe off, LHS: left heel strike, LTO: left toe off.

**Fig 6:**
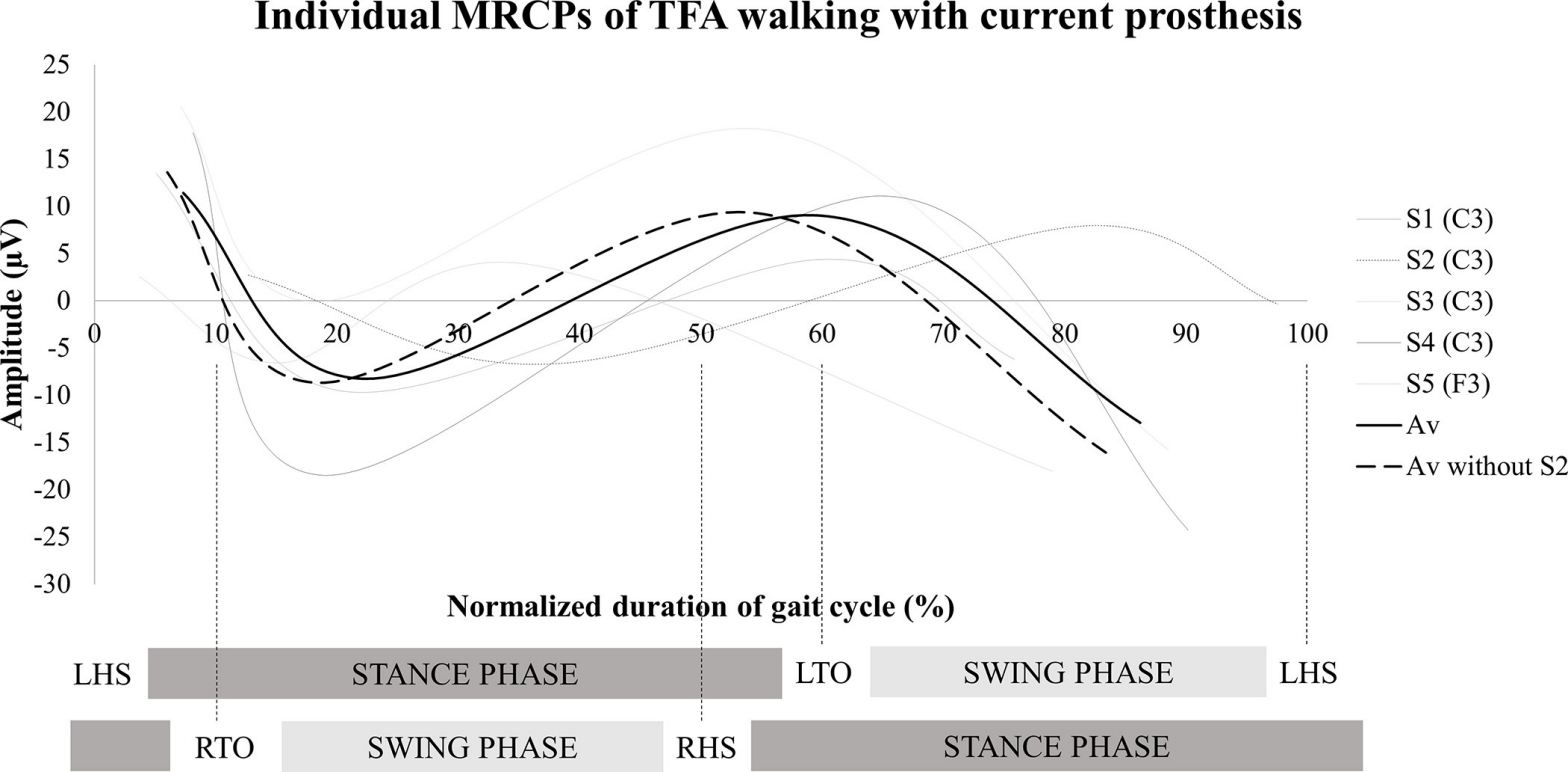
Displays a smoothed scatterplot showing individual EEG waveforms (at C3 or F3) during a gait cycle of TFA walking with current prosthetic device. **The black line represents the average of the 5 TFA. S2 is the only TFA with left amputation and therefore the dashed line represents the average of 4 TFA with right amputation. An example of gait cycle phases and events is shown below the graph (here: force-sensing resistor placed in left insole).** RHS: right heel strike, RTO: right toe off, LHS: left heel strike, LTO: left toe off.

No significant differences were observed for MRCP amplitude and latency measures at electrode Cz between able-bodied individuals (n = 6) and TTA (n = 6) walking with the current (g latency: P1 = 0.03, N1 = 0.1, P2 = 0.2 and N2 = 0.6; g amplitude: P1 = 0.9, N1 = 0.3, P2 = 0.8 and N2 = 0.4) or novel prosthetic device (g latency: P1 = 0.7, N1 = 0.1, P2 = 0.7 and N2 = 0.8; g amplitude: P1 = 0.5, N1 = 0.2, P2 = 0.2 and N2 = 0.2) (Fig 5). TFA walking with AMPfoot did not exhibit MRCPs, but TFA walking with the current prosthesis showed MRCPs at different electrode locations, i.e. C3 (n = 4), F3 (n = 1) or not at all (n = 1). Thus, no comparisons were made for MRCP amplitude and latency measures of the TFA group (Fig 6, Table 1). Fig 6 shows the inter-individual variability in cortical activity (S2 is the only left amputated subject in TFA subject group).

**Table 1:** Presents the latency (ms) and amplitude (μV) data of the different peaks/deflections of MRCPs of the different subject groups. (Note that data of able-bodied individuals and TTA are retrieved from Cz, and data of TFA mainly from C3).

	P1	N1	P2	N2
Able-bodied (n = 6)—Latency	121 ± 72	340 ± 101	614 ± 93	804 ± 77
Able-bodied (n = 6)—Amplitude	4.1 ± 1.7	-7.0 ± 4.0	3.3 ± 1.1	-6.4 ± 4.7
TTA current (n = 6)—Latency	125 ± 73	353 ± 108	639 ± 84	852 ± 66
TTA current (n = 6)—Amplitude	11.3 ± 10.7	-9.0 ± 10.0	4.8 ± 2.4	-10.2 ± 10.2
TTA AMPfoot (n = 6)—Latency	71 ± 68	329 ± 88	552 ± 65	881 ± 100
TTA AMPfoot (n = 6)–Amplitude	7.1 ± 7.5	-7.5 ± 1.8	4.1 ± 4.7	-5.6 ± 3.2
TFA current (n = 5)—Latency	73 ± 34	221 ± 91	595 ± 164	862 ± 88
TFA current (n = 5)—Amplitude	11.4 ± 8.4	-8.3 ± 6.7	9.1 ± 5.8	-12.9 ± 9.6

**Fig 7:**
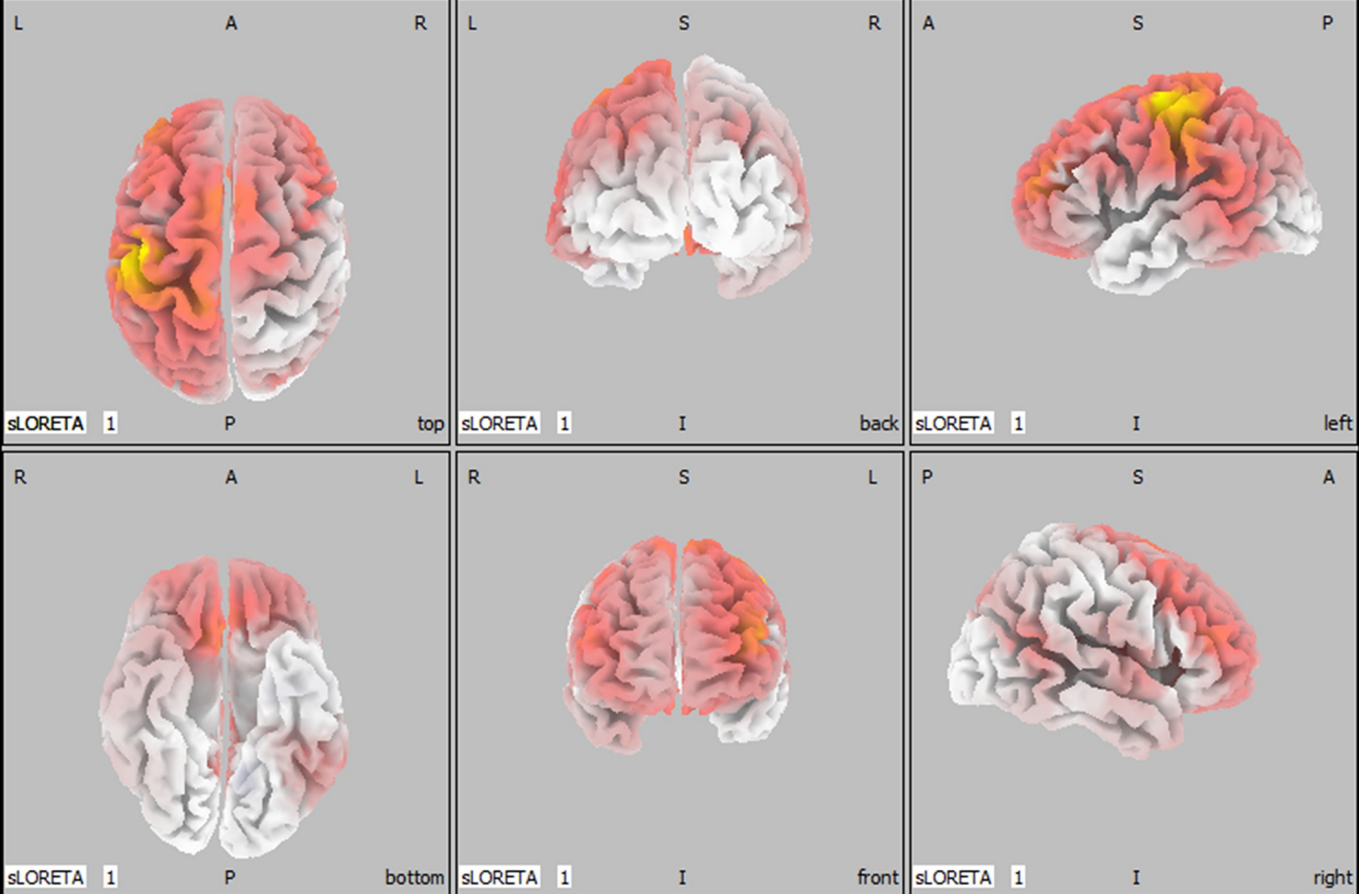
Orthogonal views of higher activity (coloured in yellow and red) of brain sources of MRCP P1 when TTA walked with AMPfoot compared to able-bodied individuals (L: left, R: right, A: anterior, P: posterior, I: inferior, S: superior). Force-sensing resistors were placed under the right foot, explaining the higher activity at P1 (phase between weight acceptance and single-limb support during gait) at the left hemisphere of the brain.

No significant differences in activity of the brain sources of the different MRCP peaks were observed when TTA walked with the current and novel prosthetic device. Additionally, no significant differences were observed when TTA walked with the current prosthetic device compared to able-bodied individuals. On the other hand, compared to able-bodied individuals TTA wearing the AMPfoot showed significant higher activity of brain sources at the first positive deflection as shown in Table 2 and Fig 7.

**Table 2:** Brain areas showing significant higher brain activity at P1 (t_critical_: 4.251) (2-tailed, p<0.05) for TTA walking with AMPfoot compared to able-bodied individuals.

Brain structure	Lobe	Brodmann area	Voxel number	Maximal MNI coordinates (X, Y, Z)	Highest voxel value
Precentral gyrus	Frontal lobe	4,6	14	40, -15, 65	5.52
Postcentral gyrus	Parietal lobe	1,2,3,40	34	-50, -20, 60	5.30
Cingulate gyrus	Limbic lobe	31	1	-20, -30, 40	5.03
Inferior parietal lobule	Parietal lobe	40	1	-50, -30, 50	4.56

## Discussion

The current study investigated neural dynamics during walking at normal, comfortable speed in healthy subjects and subjects with a transtibial or transfemoral amputation using EEG and dual tasks. We hypothesized that subjects with an amputation would require a higher attentional demand during walking compared to able-bodied individuals and that walking with the current prosthesis requires less attentional demand compared to walking with the novel prosthetic device. The attentional demand in terms of accuracy and reaction time was negatively influenced when TFA walked with AMPfoot, whereas TTA did not show any cognitive change. Thus, for TTA the hypothesis was rejected. We also hypothesized that electro-cortical activity would alter when TTA and TFA walked at normal, comfortable speed compared to able-bodied individuals. Indeed, the current study supports the altered neural dynamics in TFA because MRCPs were extracted from other electrode sites than Cz during walking with the current prosthesis. However, no MRCPs were found when TFA walked with the novel prosthesis. In TTA higher activity within brain sources of the first positive deflection of the MRCP was apparent (as shown in Fig 7).

A strong involvement of the brain during walking is fairly documented [20,21]. Cognition, in particular attention and executive functioning, significantly contributes to postural control and motor control [22]. Therefore, attentional demands during gait were explored using dual-tasks. The cognitive task, Sustained Attention to Response Task, is a sustained attention task, wherein subjects are required to overcome the tendency to respond in an automatic, task-driven manner in response to infrequent no-go stimuli. This sustained attention task was chosen since many activities of daily living require continuous attention. Although no significant differences of reaction time and accuracy were observed in TTA, attentional demands during walking were significantly elevated when TFA walked with AMPfoot compared to the current prosthesis and able-bodied individuals. It is acknowledged that risk of falling is associated with weaker gait performance as well as structural brain changes and diminished cognitive functions [23]. Brain areas involved in attention failures during the Sustained Attention to Response Task are the anterior cingulate and prefrontal cortices [24,25]. These brain areas are also involved in walking; the anterior cingulate cortex plays a role in a variety of autonomic functions, and the prefrontal cortex is closely connected with motor cortices and involved in attention and executive functions.

Neural dynamics were further explored using EEG MRCPs. MRCPs are used to predict the upcoming movement and, consequently, the identification of human movement intention has an implication in the control of external devices and assists in the development of brain-machine interface applications. Extraction of time-locked EEG segments (representing gait cycles) using heel strike has been previously used in experiments on brain dynamics during walking [26,27,28]. A previous experiment of Knaepen et al. [7] examined the electro-cortical activity during treadmill walking in healthy subjects and they observed MRCP patterns over the cortical leg representation area. Persons with an amputation face deafferentation of nerves followed by reorganization of the primary somatosensory area of the cortex [29]. The current study supports the altered neural dynamics in TFA because we were unable to extract MRCPs at the common electrode site Cz. MRCP deflections were more apparent at different electrode locations (F3 and C3). The reorganization of cortical structures is closely linked to the distorted gait patterns of TFA. The steep slope from P1-N1 (Fig 6) might be related to the longer stance phase of the intact side and longer swing phase on prosthetic side, and thus is also related to the distorted gait of TFA. On the other hand, the altered electro-cortical activity has not been observed in TTA. No significant differences in MRCP amplitudes and latencies between TTA and able-bodied individuals were detected. Although the electro-cortical activity did not change, standardized low resolution brain electromagnetic tomography revealed that TTA walking with AMPfoot 4.0 significantly increased the activity within the precentral, postcentral and cingulate gyrus, as well as the inferior parietal lobule during the first positive deflection, i.e. the toe-off phase. Thus, TTA require more neural input to accomplish the same physical activity compared to able-bodied individuals at toe-off phase.

The highest positive deflection of MRCP during the gait cycle was found at the moment where postural control during single limb support is crucial to ensure that the opposite leg is cleared to move and can be swung forward [27]. This is in accordance with Wieser et al. [28], who observed the highest cortical activity shortly before both legs are at maximal position (flexion/extension). Previous research of Wieser et al. [28] and Koeneke et al. [30] noticed that the process of changing the movement direction from extension to flexion demands the highest neural control within the involved cortical network, i.e. the motor cortices. Important to note is that the electrode Cz is located on top of the primary motor cortex and more specifically overlies the leg representation area of the classical sensory and motor homunculus [31]. Multi-sensory integration of information coming from somatosensory, visual and vestibular sensation act on different brain areas, i.e. cingulate, premotor and prefrontal cortices [32], which might result in the positive deflections P1 and P2 observed at Cz. After reaching positive peak amplitudes the electro-cortical activity returns to baseline levels. The current study shows that during the pre-swing phase or the acceleration phase electro-cortical activity progressively decreases. In this phase Brunner & Rutz [27] noted that the leg is accelerated as a bi-articular pendulum that folds and extends passively during swing. This negative deflection can be considered as preparatory phase for the most critical point of walking where postural control is crucial. If the locking system of the AMPfoot 4.0 integrates an automated detection of the (individual) negative slope of the MRCP, this feature extraction can be used to improve the accuracy of unlocking the free ankle movement during swing phase. Note that in the current study treadmill walking was used and thus, MRCP features might differ from hallway walking.

Furthermore, MRCP features differ among subjects (as clearly visualized in Figs 5 and 6), which is also caused by different walking speeds. Literature has clearly shown that walking speed influences electrocortical activity [33,34]. Although subjects walked at their normal walking speed in the current study, TFA and TTA walked 46% and 21% slower compared to able-bodied individuals.

One of the limitations of the study is that subjects with an amputation were not accustomed to the different mechanical system and weight of the AMPfoot 4.0. Normally, subjects need an acclimation period to the novel device for several months, but due to restricted financial support and availability of prototypes (1 backup device) a very short adaptation period was included in the protocol. Since neural dynamics alters in TTA and TFA compared to able-bodied individuals, future work should focus on a redesign of AMPfoot 4.0 with the integration of automated electro-physiological feature extraction coupled with the locking system of AMPfoot 4.0 to improve the control of movement of the relevant mechanical components involved in propulsive forces. Another limitation of the study is that data were normalized, but this approach might express reduced peak magnitudes and increased standard deviations due to inter- and intra-cycle variability in timing [35, 36].

In Brain Computer Interface applications, it is of utmost importance to extract brain information and to remove artifacts. In literature, there is current debate about the removal of gait-related movement artifacts. Frequency bandwidths similar to MRCPs are prone to low frequency motion artifacts (and EOG). Spatial filtering is commonly applied for artifact removal and for improving detection accuracy of cortical potentials [37]. One of the most common spatial filters used in EEG-based BCI systems that showed to be successful in artifact reduction and source localization, is ICA [38]. The current study demonstrated MRCPs with amplitudes within the normal range and using inverse-based modeling we showed brain activity within brain areas where we would expect brain activity during walking (i.e. sensorimotor and parietal cortices; cfr [39], [40]). In the current paper, active electrodes were applied. These electrodes are less susceptible to sudden changes in voltage compared to passive electrodes, which are more susceptible to sudden changes in impedance and capacitance [41]. However, it still remains arguable whether all artifacts were removed. Indeed, current existing EEG techniques and data cleaning methods during gait are still limited, and in literature there is a debate as to how neural data containing gait-related movement artifacts is separated by ICA. An interesting approach for movement artifact removal is to simultaneous measure movement artifact and movement artifact plus neural signal, because this method allows for an interpolative subtraction process that could remove the movement artifact [42]. Accelerometer placement on the forehead is not recommended, since small movements of electrodes relative to a subject cause the electrodes to register a change in voltage, whereas the accelerometer only measures changes in the head’s acceleration relative to the environment [41]. Kline et al. [41] and Snyder et al. [42] clearly outlined that the integration of multiple algorithms with ICA could allow for additional artifact identification and rejection, especially at faster walking speeds. More research is required on the combination of different methods to establish the optimal method for distinguishing neural data from artifact in EEG during human walking.

To conclude, subjects with an amputation above the knee reduced attentional demands and altered the electro-cortical activity during walking at normal, comfortable speed with AMPfoot 4.0. Subjects with an amputation below the knee showed increased activity within underlying brain sources of the first positive MRCP deflection at toe-off phase indicating a compensatory mechanism to maintain postural control during walking. An automated extraction of the negative slope of the MRCP coupled with the locking system of the AMPfoot 4.0 might improve the mechanism involved in the propulsive force transmission.

## Supporting information

S1 File Excel file containing EEG and dual task data. (XLSX)

S2 File Zipped file with dual-task datasets. (ZIP)

S3 File Zipped file containing raw EEG datasets of able-bodied individuals (Part 1). (ZIP)

S4 File Zipped file containing raw EEG datasets of able-bodied individuals (Part 2). (ZIP)

S5 File Zipped file containing raw EEG datasets of individuals with a transfemoral amputation (TFA) walking with the current prosthesis (Part 1). (ZIP)

S6 File Zipped file containing raw EEG datasets of individuals with a transfemoral amputation (TFA) walking with the current prosthesis (Part 2). (ZIP)

S7 File Zipped file containing raw EEG datasets of individuals with a transfemoral amputation (TFA) walking with the novel prosthesis (Part 2). (ZIP)

S8 File Zipped file containing raw EEG datasets of individuals with a transfemoral amputation (TFA) walking with the novel prosthesis (Part 1a). (ZIP)

S9 File Zipped file containing raw EEG datasets of individuals with a transfemoral amputation (TFA) walking with the novel prosthesis (Part 1b). (ZIP)

S10 File Zipped file containing raw EEG datasets of individuals with a transtibial amputation (TTA) walking with the current prosthesis (Part 1). (ZIP)

S11 File Zipped file containing raw EEG datasets of individuals with a transtibial amputation (TTA) walking with the current prosthesis (Part 2). (ZIP)

S12 File Zipped file containing raw EEG datasets of individuals with a transtibial amputation (TTA) walking with the current prosthesis (Part 3). (ZIP)

S13 File Zipped file containing raw EEG datasets of individuals with a transtibial amputation (TTA) walking with the novel prosthesis (Part 1). (ZIP)

S14 File Zipped file containing raw EEG datasets of individuals with a transtibial amputation (TTA) walking with the novel prosthesis (Part 2). (ZIP)

S15 File Zipped file containing raw EEG datasets of individuals with a transtibial amputation (TTA) walking with the novel prosthesis (Part 3). (ZIP)
